# When is enough enough? Empirical guidelines to determine participant sample size for scene viewing studies

**DOI:** 10.3758/s13428-025-02754-8

**Published:** 2025-07-28

**Authors:** Alex J. Hoogerbrugge, Ignace T. C. Hooge, Roy S. Hessels, Christoph Strauch

**Affiliations:** Experimental Psychology, Helmholtz Institute, Utrecht University, Utrecht, The Netherlands

**Keywords:** Eye tracking, Sample size, Power estimation, Heatmap, Gaze, Area of interest

## Abstract

Eye tracking is widely used to study where spatial attention is allocated across stimuli. However, determining a sufficient and efficient number of participants for such studies remains a challenge. While clear guidelines have been established for many classical statistical tests, no straightforward participant sample size guidelines exist for the comparison of gaze distribution maps and area-of-interest analyses – two of the most prominent analyses in scene viewing studies. Just how many participants should be included for reliable and reproducible gaze estimations? We here utilized gaze data to a single static image, viewed by 1248 individuals (dataset 1), and gaze data to 200+ images, viewed by 84 participants each (dataset 2). Researchers can assess which of these datasets and analysis types most resemble their setup and determine their sample size accordingly. Although we cannot provide a one-size-fits-all sample size recommendation, we show progressively diminishing returns for a range of sample sizes and for two typical study types. For example, when using Normalized Saliency Score as a metric of distribution map similarity, a 5% relative increase requires increases in sample size from 13 $\rightarrow$ 20 $\rightarrow$ 34 participants (based on dataset 1) or from 10 $\rightarrow$ 16 $\rightarrow$ 32 participants (based on dataset 2). Alternatively, when analyzing the number of visits to certain areas of interest, a 25% decrease in outcome variance requires increases in sample size from 13 $\rightarrow$ 24 $\rightarrow$ 44. We provide easy-to-use guidelines and reference tables to determine scene viewing participant sample size for academics and industry professionals alike.

**Supplementary Information:**

The online version contains supplementary material available at 10.3758/s13428-025-02754-8.

## Introduction

In many fields, researchers are interested in where people look – or better yet, where people *will* look. For example, analyses of gaze behavior during scene viewing are frequently used in psychological research to investigate how certain image features relate to spatial attention (Itti et al., 1998; De Haas et al., 2019; Tatler et al., 2011). In applied and commercial settings, similar methods are often used to, e.g., analyze the gaze behavior of pilots in cockpits, or to predict where people will look on a marketing billboard (Bylinskii et al., 2017; Ziv, 2016).

**Fig. 1:**
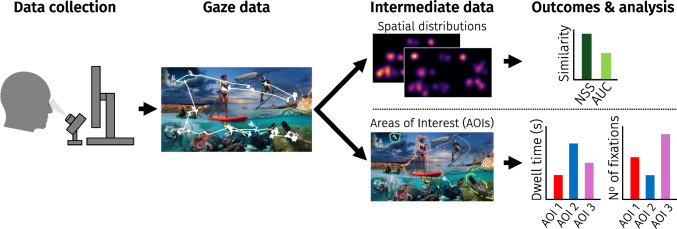
Schematic overview of a typical scene-viewing study. *Data collection:* Participants are shown one or multiple images consecutively while their eye movements are tracked. *Gaze data:* The eye tracker signal is converted into gaze data, expressed as *x*- and *y*-coordinates over time. We here present the stimulus used in dataset 1, overlaid with an example gaze trace in white. *Intermediate data:* After all data is collected, gaze coordinates are converted to intermediate data. For this, raw gaze samples can be used directly from the eye tracker, or fixations can be extracted first. Gaze data can be converted to *spatial distribution maps*, in which gaze data is converted into a map of *x*- and *y*-coordinates; time is often ignored. Gaze data can also be linked to *areas of interest* (AOIs, outlined in color); these can be programmatically or manually defined. *Outcomes & analysis:* The similarity of spatial distributions from (sub-)samples of participants can be calculated using, e.g., normalized saliency score (NSS) or area under the curve (AUC). AOIs can be compared in terms of, e.g., the dwell time or the number of fixations within each AOI

After gaze data collection, gaze locations obtained during scene viewing are often first transformed into a spatial distribution map (hereafter: distribution map). These distribution maps are usually of the same size as the stimulus material (e.g., 1920 $\times$ 1080 pixels), in which the values at pixels indicate how many gaze samples were recorded at those locations. In turn, distribution maps are most frequently visualized as heatmaps, with color to represent the values of pixels (see e.g., Fig. 1). Because heatmaps are simply the visualization of distribution maps, it is probably easiest to henceforth imagine a heatmap whenever we mention distribution maps. Distribution maps form the basis for quantifying how participants distribute their attention across a given stimulus. One way to analyze these maps is in a content-agnostic manner, for example by comparing distribution maps in their totality to reference data or to predictive models, using Normalized Scanpath Saliency (NSS), Area Under the Curve (AUC), or similar metrics. These values provide an estimate of the (dis)similarity between distribution maps (Cristino et al., 2010; Lao et al., 2017; Kümmerer et al., 2018; Riche et al., 2013). Alternatively, researchers may be interested in how gaze is allocated to specific areas of interest (AOIs; Orquin et al., 2016; Vehlen et al., 2022; Hessels et al., 2016). An AOI is a region within the visual stimulus (e.g., Fig. 1) that the researcher intends to examine based on the corresponding eye tracking data (Holmqvist et al., 2011, Chapter 6, p. 187). Researchers from various fields (e.g., science, art history, marketing research, psychology, cartography, radiology, traffic safety) use AOIs to study the spatial and temporal looking behavior of their participants in relation to a given visual stimulus (e.g., advertisement, painting, mammogram, map of France). By analyzing AOIs, researchers can determine which part of the stimulus attracts the most attention, which aspects of a map are ignored, or in what sequence a webpage is viewed.

How many participants should one include in their study to generate a reliable and replicable estimate of the gaze distribution across a stimulus? Funding agencies, journals, ethics boards, customers, or project managers usually require researchers to determine the sample size that will be used *before* a study starts. However, one does not want to waste valuable money, time, and effort (both of participants and researchers) on collecting unnecessarily large samples. Choosing an appropriate sample size thus requires researchers to trade-off between maximizing information gain and statistical power, while minimizing ethical and monetary costs (Morse, 2000; Lakens, 2022; OPEN Science COLLARABORATION, 2015). It is therefore highly relevant – for academics and industry professionals alike – to make informed estimations for optimal sample sizes needed to obtain a spatial distribution of gaze locations given a desired level of generalizability in relation to resource constraints.

While scene viewing studies are widely used across disciplines (e.g., Martinez-Marquez et al., 2021; Lorigo et al., 2008; Mat Zain et al., 2011), no clear guidelines for participant sample size determination currently exist. In contrast to the many guidelines and tools for determining sample size where statistical tests are involved (e.g., Browne, 1995; Adcock, 1997; Brysbaert, 2019; Faul et al., 2007), this is not straightforward for scene viewing comparisons, because the commonly used metrics (e.g., NSS, AUC) are not directly associated with effect sizes. Moreover, given relatively high inter-observer variability in scene viewing behavior (Strauch et al., 2023, 2024), pilot studies with just a few participants may not always give a good estimate of what population behavior will look like (Albers & Lakens, 2018). Therefore, the current state of sample size determination in scene viewing unfortunately still involves using rules of thumb rather than empirical guidelines. Is one participant enough? Is 500? Although we cannot answer this question with a one-size-fits-all solution, we provide researchers with reference figures and tables to select a *sufficient* yet *efficient* sample size for scene viewing studies. Depending on the use case, highly accurate estimates with minimal variance may be required (think of academic research or a safety-critical computer interface), but in other cases, a coarser estimate will suffice. We provide the means to make this informed decision here.

Specifically, we approach the matter of participant sample size determination from the perspective of *data saturation*. This term relates to the law of diminishing returns, where collecting additional data points at the same cost eventually only brings a sample’s value marginally closer to the population value (Morse, 2000). The idea of data saturation is applied in various qualitative research settings (Guest et al., 2017; Glaser & Strauss, 2017; Guest et al., 2006; Hennink et al., 2019), and can, as we argue, also help determine sample size in scene-viewing studies. Let’s say that with *n* = 3 participants, five unique areas are fixated, that with *n* = 10 participants the number of fixated areas becomes eight, and that with *n* = 30 participants this number remains eight. In hindsight, we could have stopped collecting gaze data after ten participants were reached, as saturation was achieved. With this reasoning Nielsen and Landauer (1993) showed that the number of unique operator errors encountered in human–computer interactions is highly dependent on sample size, but that the added value of each additional participant gradually diminishes (see Lewis, 2012, for further nuance and details in usability testing and human factors, especially when it comes to detectability). However, what would happen if we included data from a hundred or a thousand participants? Perhaps several new unique areas of interest are fixated when increasing sample size by such large steps. Because of this, Faulkner (2003) argued that the point of saturation cannot be discovered without first collecting a larger sample. In other words, in order to estimate what sample size is needed for a desired level of stability in scene viewing outcomes, guidelines based on large data sets are needed. To this end, we here used two such large datasets.

First, we report on an eye tracking dataset of *n* = 1248 participants of diverse ages who viewed a single, but feature-rich, image for 10 s, using an unsupervised eye tracking setup. Second, we report on an experiment in which 200+ images were viewed by at least 84 participants each, obtained in a highly controlled and supervised setting with top-of-the-line equipment (Wilming et al., 2017; Onat et al., 2014; Ossandón et al., 2014). These datasets are illustrative of two possible approaches to study scene viewing:

1. Moderate cost (thus lower quality per data point), minimal experimenter involvement (thus limited monitoring of participants and possibly more environmental distractions), low participant requirements (single image, up to 4 min of participation).
2. High cost (thus higher quality per data point), extensive experimenter involvement (thus effective monitoring of participants and more control over environment), moderate participant requirements (multiple images, up to 1 h of participation).

Due to these differences, one can expect that each additional participant will be associated with more variability (less predictability) of information gain in dataset 1 than in dataset 2. Besides providing sample size guidelines for both study types, we provide guidelines for both distribution map-based analyses and AOI-based analyses. Scene viewing researchers can assess which of the datasets and analyses presented here more closely align with their experimental setup and goals, and can choose their sample size accordingly.

**Fig. 2:**
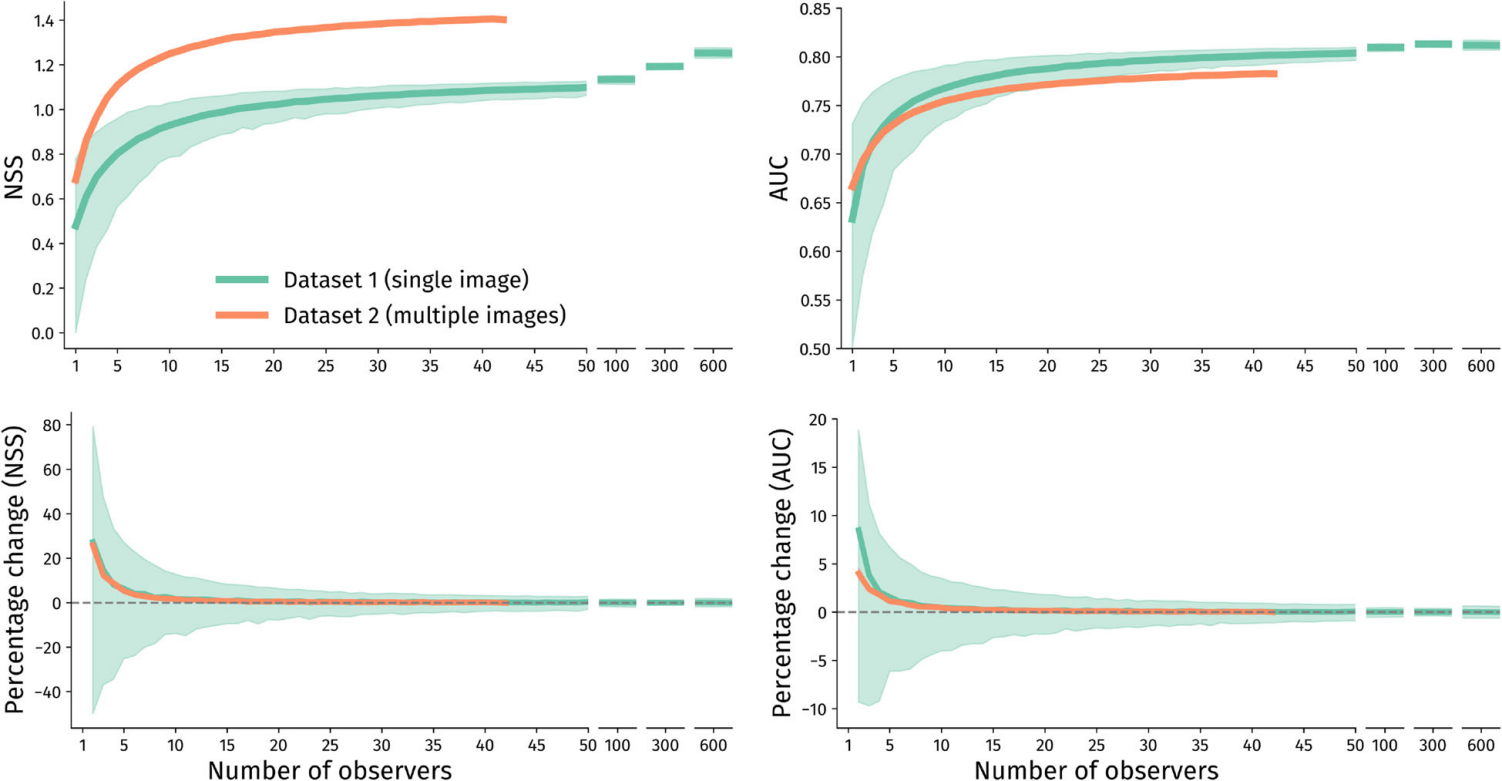
Comparisons of gaze distribution maps for different sample sizes. *Top* Distribution map similarity metrics (NSS: normalized scanpath saliency; AUC: area under the curve) as a result of sample size compared to a ’benchmark’ of all other participants. *Bottom* Percentage change in distribution map similarity metrics per additional participant (*n* compared to *n* - 1). Dataset 1 was bootstrapped 1000 times; dataset 2 was bootstrapped ten times due to lower inter-individual variability. *Solid lines* indicate the average value, *shaded areas* denote the 95% range of bootstrapped values

## Methods

Data and code are available via the Open Science Framework https://osf.io/sk4fr/.

### Distribution maps

#### Dataset 1: Single image

We analyzed gaze data of 1248 participants (note that valid demographics could only be obtained for a subset of *n* = 1188: 4–76 years old *M* = 27.4, *SD* = 12.9, 485 female, 614 male, 89 other) who viewed a single image at the NEMO Science Museum Amsterdam. For the presented stimulus, see Fig. 1, and for a high-resolution image and pictures of the setup, see Strauch et al. (2023). Note that the presently used data is new data relative to the data assessed with a less precise Tobii 4C tracker and reported on in Strauch et al. (2023). For the current setup and tracker, please see Hoogerbrugge et al. (2025). The stimulus was presented on a 27-inch, 1920 $\times$ 1080 monitor (circa 50$^\circ$ $\times$ 24$^\circ$ visual angle) with a maximum luminance of 300 cd/m^2^. The monitor was mounted inside of a metal box which partially shielded from external light and thereby kept the environmental brightness relatively constant. Participants’ gaze was tracked using a Tobii Pro Fusion at 60 Hz (Tobii, Sweden), which was positioned directly below the monitor at circa 80 cm from participants’ eyes. A five-point calibration was performed before running the experiment. Participants received no task instructions regarding the experiment except to ’take a look’, after which they were simply presented the image for 10 s. Because the stimulus was preceded by centrally placed text, participants generally gazed at the center of the screen when the stimulus was first presented. After the 10-s freeviewing period, participants were shown a replay of their gaze behavior, were asked to donate their data to research, and were asked to indicate demographic data (age and gender) if they had consented to donate their data.

Raw gaze samples were used as a basis for spatial distribution maps. To make data comparable to most scene-viewing datasets (see e.g., Kümmerer et al., 2018), we here used only the first 5 s. Results were highly similar when using fixation locations instead. To partially quantify data quality, we computed the sample-to-sample root median squared displacement over the raw gaze data (RMS-S2S; Hooge et al., 2018; Dunn et al., 2024; Niehorster et al., 2020, note that we use median instead of mean in order to ameliorate outliers from saccades). In dataset 1, the RMS-S2S was on average 0.41$^\circ$ across participants (*SD* = 0.15$^\circ$).

#### Dataset 2: Multiple images

We re-analyzed gaze data of 115 participants (19–28 years old) who viewed 249 static images of natural scenes, urban scenes, fractal images, and pink noise images. These data were obtained from Wilming et al. (2017) (specifically, we used the Baseline, Bias, and Gap datasets) and are described in more detail in Onat et al. (2014); Ossandón et al. (2014). The images were 1280 $\times$ 960 pixels (circa 28$^\circ$ $\times$ 22$^\circ$ visual angle) and were viewed for at least 5 s per participant. Gaze data was recorded with an EyeLink II eye tracker at 500 Hz, and we used fixation data to generate spatial distribution maps. Each image was viewed by at least 84 participants. As for the first dataset, we used the first 5 s of freeviewing, now for each image. The RMS-S2S of the raw gaze data was on average 0.006$^\circ$ across participants (*SD* = 0.003$^\circ$).

#### Comparing spatial distribution maps for different sample sizes

To assess the generalizability of any given distribution map, it needs to be compared to a larger sample, which we here denote as a *benchmark*. We used a leave-n-out procedure to produce this benchmark: We computed distribution maps for sample sizes between *n* = 1 and *n* = 624 (half of participants in dataset 1) or *n* = 42 (half of participants in dataset 2), and used the combined distribution maps of the respective remaining halves to serve as benchmarks. We then computed NSS and AUC (see below) to assess how representative each sample size *n* was compared to its benchmark. To map possible effects from picking ’(un)lucky’ samples, we repeated this procedure 1000 times (dataset 1) or 10 times (dataset 2), each time taking a random sample of participants of size *n* (i.e., bootstrapping).

There are several metrics that can be used to compare the similarity or dissimilarity between distribution maps, of which we here used normalized scanpath saliency (NSS) and area under the curve (AUC). We chose these metrics for their popularity, but note that, in practice, these two metrics and many others tend to correlate closely (Bylinskii et al., 2018; Le Meur & Baccino, 2013; Kümmerer et al., 2018).

NSS is relatively sensitive to subtle differences between distribution maps, but has the disadvantage of having no upper bound and being somewhat sensitive to increasing as sample size increases (Riche et al., 2013). NSS was computed as in Peters et al. (2005); Le Meur and Baccino (2013). Specifically, we took the combined gaze data of a selected sample as our comparison distribution map, which was Gaussian blurred with a sigma of 1$^\circ$ visual angle (circa 45 $\times$ 45 pixels) and standardized (i.e., z-scored). The distribution map of all other participants was considered the benchmark distribution map, which was standardized but not blurred. We then used the benchmark distribution map as a mask, and calculated the mean of all values from the comparison distribution map in locations where the benchmark distribution map received more than an average amount ($z \ge 0$) of fixations. This mean value constitutes the NSS value. A higher NSS indicates higher overlap between two distribution maps.

AUC scores differ from NSS, in that they are bounded between 0 and 1. They are therefore more comparable across datasets and are less sensitive to differential sample sizes. Perfect correspondence between two distribution maps is indicated with AUC = 1 and chance level with AUC = 0.5 (but note that perfect correspondence practically rarely occurs; see Zhao & Koch, 2011). We calculated AUC by first standardizing (z-scoring) the benchmark distribution map and setting all pixels where $z \ge 0$ as true and $z < 0$ as false. We then Gaussian blurred and scaled the values in our comparison distribution map between 0 and 1, which we considered ’predictions’. Using these benchmark and prediction arrays, the AUC could be computed as normal (e.g., Le Meur & Baccino, 2013).

### Area-of-interest-based measures

**Fig. 3:**
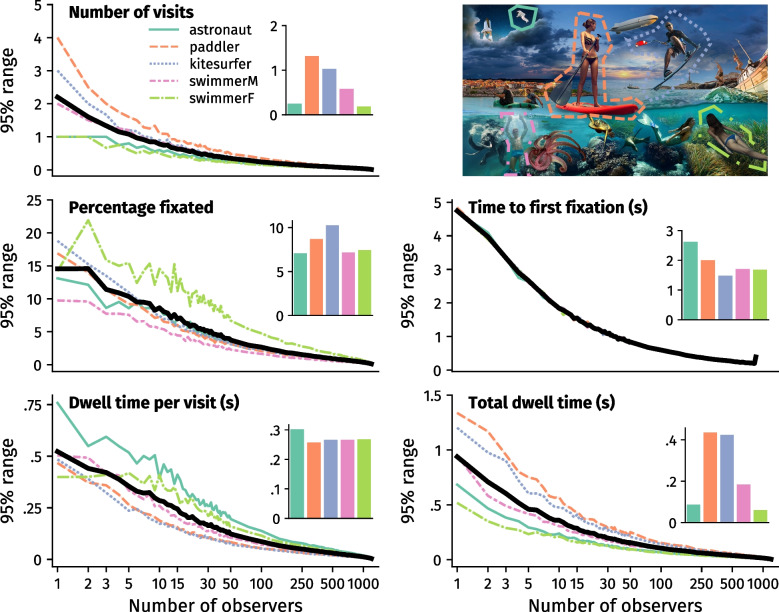
*Top right*: The image used in dataset 1, overlaid with a selection of five areas of interest (AOIs). *Other panels*: Variance of AOI fixation outcomes as a function of sample size. *Inset bar* plots show the average values over which the variance was calculated. Variance is expressed as the 95% range of values obtained from bootstrapping the outcomes for each AOI 1000 times (effectively establishing a 95% confidence interval). Note that the *x*-axis is on a log scale. *Colored lines* denote individual AOIs, the *solid black line *denotes the average across AOIs

To determine how many participants are needed for AOI-based analyses, we analyzed data only from dataset 1 (Strauch et al., 2023). Instead of raw gaze samples, we used fixation data of the first 5 s of scene viewing, classified using the I2MC algorithm in Python (v2.2.4; Hessels et al., 2017). AOIs were manually defined by outlining several key objects within the image (see Fig. 3 upper right). These AOIs were hand-drawn to reflect differently large AOIs, different eccentricities, different saliency levels, etc. The (absence of) differences across AOIs can therefore also inform about how comparable findings are across different types of AOIs. Furthermore, minimal axes of AOI were never smaller than 2.2°, conforming to recommendations on AOI size (Orquin & Holmqvist, 2018; Holmqvist et al., 2011). We refer the interested reader to Orquin et al. (2016); Holmqvist et al. (2011) for more information on how to optimally draw AOIs and minimal sizes (scaling with eye tracker accuracy), be it by hand or by using algorithmic approaches (e.g. Ravi et al., 2024). For each AOI, we computed: (1) the number of visits to that AOI, separated by at least one fixation outside of the AOI; (2) the percentage fixated, i.e., the number of fixations within the AOI expressed as a percentage of the total number of fixations made during scene viewing; (3) Time to first fixation within the AOI (in seconds); (4) Dwell time per visit (s), i.e., the total fixation duration within the AOI divided by the number of visits; (5) Total dwell time (s), computed as the sum of fixation durations within the AOI.

**Table 1:** Sample size required for 5% relative increase (NSS and AUC) from the previous sample size, starting at *n* = 1

Dataset 1 (single image)				Dataset 2 (multiple images)			
*n*	AUC	*n*	NSS	*n*	AUC	*n*	NSS
1	0.63	1	0.48	1	0.67	1	0.68
2	0.69	2	0.61	3	0.71	2	0.86
4	0.73	3	0.70	8	0.75	3	0.97
10	0.77	4	0.75	> 42	0.78	4	1.05
± 100	0.81	5	0.80			5	1.11
> 624	0.85	7	0.87			7	1.18
		9	0.91			10	1.25
							
							
		34	1.07			> 42	1.46
		± 100	1.13				
		± 300	1.19				
		± 600	1.25				
		> 624	1.31				

Note that after *n* = 50 we provide estimates on the order of 100 participants. Highlighted in  : When using a typical *lower*-cost setup, in order to increase the generalizability by 5% from 13 participants, one would need to collect data from an additional seven participants (when using NSS as a comparison metric). Highlighted in  : When using a typical *higher*-cost setup, in order to increase the generalizability by 5% from 16 participants, one would need to collect data from an additional 16 participants (when using NSS as a comparison metric)

**Table 2:** Cross-reference table

	

NSS and AUC percentage change between sample size increments in dataset 1 (single image). Highlighted in  : When using NSS as a metric, one can expect a 6.8% relative increase when measuring data from 15 instead of ten participants, or a 14.6% relative increase when measuring data from 30 instead of ten participants. Highlighted in  : When using AUC as a metric, one can expect a 1.8% relative increase when measuring data from 15 instead of ten participants, or a 3.8% relative increase when measuring data from 30 instead of ten participants

These metrics were computed for sample sizes ranging from *n* = 1 to *n* = 1200. Again, to map and ameliorate possible effects from picking ’(un)lucky’ samples, we bootstrapped this procedure 1000 times, each time taking a random sample of participants of size *n*.

## Results

### Distribution maps

#### Dataset 1: Single image

In Fig. 2 (green lines) we report, for sample sizes from *n* = 1 to *n* = 600, the NSS and AUC scores compared to the benchmarks, including 95% ranges of the 1,000 bootstrap iterations (effectively establishing 95% confidence intervals). We further report percentage changes in NSS and AUC between consecutive sample sizes (*n* versus *n* - 1). These data show that the biggest information gain per added participant is achieved at smaller sample sizes, up to circa *n* = 15. After this, the information gain for each additional participant asymptotes (i.e., flattens out), and any sample sizes larger than approximately *n* = 40 yield around 0% gain per added participant, irrespective of the outcome metric used.

However, one should keep in mind that these are averages, and that (due to inter-observer variability) even going from *n* = 40 to *n* = 41 can bring the distribution map several percentage points closer to – or further away from – the benchmark. We therefore report in Table 1 the sample sizes required to achieve, on average, 5% increases in NSS and AUC, respectively. For example, with *n* = 13 participants, the NSS in dataset 1 is 0.97. To achieve a 5% higher NSS (= 1.02), one should increase their sample size to circa *n* = 20 (highlighted in green, Table 1).

Lastly, we report a reference table (Table 2) of the expected percentage change in NSS and AUC for larger increments in sample size. For example, one can expect a 6.8% relative improvement in NSS when measuring 15 instead of ten participants, or a 14.6% relative improvement in NSS when measuring 30 instead of ten participants (highlighted in yellow, Table 2). When using AUC, these values are smaller; 1.8% and 3.8% respectively (highlighted in blue, Table 2).

#### Dataset 2: Multiple images

In Fig. 2 (orange lines) we report, for sample sizes from *n* = 1 to *n* = 42, the NSS and AUC scores compared to the benchmarks, including 95% ranges of the 1000 bootstrap iterations. Although NSS and AUC scores are higher and slightly lower, respectively, compared to data from a single image, the overall patterns of added value per participant are highly comparable between the two datasets. Notably, variance almost completely disappears compared to dataset 1 due to using many images per participant. This means that each added participant in dataset 2 will more reliably improve the generalizability as compared to dataset 1.

**Table 3:** Cross-reference table

	

NSS and AUC percentage change between sample size increments in dataset 2 (multiple images). Highlighted in  : When using NSS as a metric, one can expect a 5% relative increase when measuring data from 15 instead of ten participants, or a 10.6% relative increase when measuring data from 30 instead of ten participants. Highlighted in  : When using AUC as a metric, one can expect a 1.4% relative increase when measuring data from 15 instead of ten participants, or a 3.1% relative increase when measuring data from 30 instead of ten participants

In Table 1 we report the sample sizes required to achieve, on average, 5% increases in NSS and AUC. For example, with *n* = 16 participants, the NSS in dataset 2 is 1.32. To achieve a 5% higher NSS (= 1.39), one should increase their sample size to circa *n* = 32 (highlighted in orange, Table 1).

In Table 3, we report the expected percentage change in NSS and AUC for larger increments in sample size. For example, one can expect a 5.0% relative improvement in NSS when including 15 instead of ten participants, or a 10.6% relative improvement in NSS when including 30 instead of ten participants (highlighted in yellow, Table 3). When using AUC, these values are smaller; 1.4% and 3.1% respectively (highlighted in blue, Table 3).

How generalizable are these findings across image categories (fractals, urban, natural, pink noise)? We reran analyses for dataset 2 per image category. While there was some variation between categories, this variation was limited overall. For instance, at a sample size of *n* = 15 observers, an additional *n* = 5 observer yielded an overall NSS improvement of 2.8% to 3.4% (natural scenes: 2.8%, urban scenes: 2.1%, fractals: 2.5%, pink noise: 3.4%; see Supplementary Figure 1 and Supplementary Tables 1-5 for full results for both NSS and AUC). This lets us conclude that findings/guidelines are generalizable across image categories.

### Area-of-interest-based measures

Although averaged AOI metrics differed between the five reported AOIs, due to the bootstrapping procedure, there were limited differences in averaged metrics between, e.g., *n* = 1 or *n* = 1200. However, the estimated possible values that the average could take differed considerably between different sample sizes. For example, with a random sample of ten participants, we may find that the ’paddler’ AOI was on average visited 1.21 times per participant. From the other 999 iterations in our bootstrapping procedure, though, we could estimate that with any different sample of ten participants, this average value would be likely to range anywhere between 0.7 and 1.8 visits (i.e., 95% range; Fig. 3 top left). With a random sample of 100 participants, we found an average of 1.23 ’paddler’ AOI visits – which is highly similar to the 10-participant average – but we could estimate that any other sample of 100 participants would be likely to average between 1.07 and 1.42 visits. For any 1000 participants, the average is still 1.23, but with an estimated range of 1.21 to 1.26 visits for any other equally large sample. As such, larger sample sizes allow us to make a narrower range of estimates for a population average.

In Fig. 3 we report the 95% ranges of bootstrapped values for each AOI metric, on a selection of five AOIs (Fig. 3 top right). Specifically, the 95% range denotes the 97.5^th^ percentile minus the 2.5^th^ percentile of bootstrapped values. These reported ranges provide an indication of how much variance in AOI metrics can be expected when selecting various sample sizes. It becomes clear from Fig. 3 that, with smaller sample sizes, the to-be-expected variance in data was quite high, but decreased as sample size increased, eventually converging toward 0.

In Table 4 we report the sample sizes required to decrease this variance by 25%, relative to the previous sample size. For example, the to-be-expected variance in the number of AOI visits decreased by 25% between sample sizes 1, 3, 7, 13, 24, etc. (highlighted in yellow, Table 4).

In Table 5 we report the change in variance between larger increments of sample sizes. For example, when analyzing the *number of AOI visits*, we found a 18.1% relative decrease in variance when measuring data from 15 instead of ten participants, or a 40.7% relative decrease when measuring data from 30 instead of ten participants (highlighted in yellow, Table 5). The variance in *dwell time per visit* decreased by 19.2% and 42.7% from 10 to 15 and 30 participants, respectively (highlighted in blue, Table 5).

**Table 4:** Sample size required for 25% relative decrease in variance from the previous sample size when analyzing AOI-based metrics, starting at *n* = 1

Number of visits	Percentage fixated	Time to first fixation	Dwell time per visit	Total dwell time
	1	1		1
	4	3		3
	10	6		6
	20	11		11
	36	20		21
44	± 75	37	± 75	36
± 75	± 150	± 75	± 125	± 75
± 150	± 250	± 150	± 225	± 150
± 275	± 400	± 275	± 375	± 250
± 425	± 575	± 500	± 550	± 400
± 600	± 775	> 1200	± 750	± 575
± 775	± 950		± 925	± 750
± 950	± 1050		± 1050	± 925
± 1075	± 1150		± 1125	± 1050
± 1150	± 1200		± 1175	± 1125
± 1200	> 1200		> 1200	± 1175
> 1200				> 1200

Note that after *n* = 50 we provide estimates on the order of 25 participants. Estimates are from dataset 1. Highlighted in  : When analyzing the number of AOI visits, variance is expected to decrease by 25% between sample sizes 1, 3, 7, 13, 24, etc. Highlighted in  : When analyzing the dwell time per AOI visit, variance is expected to decrease by 25% between sample sizes 1, 5, 10, 18, 33, etc.

**Table 5:** Cross-reference table

	

Percentage change in variance (95% Confidence Intervals) between sample size increments in dataset 1. Highlighted in  : When using the number of AOI visits as a metric, one can expect a 18.1% relative decrease in variance when measuring data from 15 instead of ten participants, or a 40.7% relative decrease when measuring data from 30 instead of ten participants. Highlighted in  : When using the dwell time per AOI visit as a metric, one can expect a 19.2% relative decrease in variance when measuring data from 15 instead of ten participants, or a 42.7% relative decrease when measuring data from 30 instead of ten participants

## General discussion

Analyses of gaze behavior during scene viewing are frequently used across disciplines (Itti et al., 1998; Bylinskii et al., 2017; Ziv, 2016). As in other research fields, having a well-substantiated participant sample size is important in order to achieve *sufficient* statistical power at the highest *efficiency*. However, common analysis methods for scene viewing (i.e., gaze distribution comparisons) are not directly associated with existing power analysis methods, and therefore no guidelines for determining participant sample size in scene viewing have been available thus far. We addressed this here, and provide practical guidelines based on two large datasets. Which sample size is optimal depends on the research question at hand, as well as on the experimental setup and researchers’ resource constraints, among others (e.g., Morse, 2000). Although we cannot make a one-size-fits-all recommendation, dataset 1 was used to represent a typical lower-cost and lower experimental control data collection method, whereas dataset 2 represents a typical high-end lab study. We additionally addressed two of the most frequently used analysis methods, namely distribution map comparisons and AOI-based analyses.

We showed that scene viewing studies are subject to the law of diminishing returns (i.e., saturation), as is the case in other types of research (Guest et al., 2017; Glaser & Strauss, 2017; Guest et al., 2006; Hennink et al., 2019; Nielsen & Landauer, 1993; Faulkner, 2003; Morse, 2000). Notably, the added value per participant was, on average, quite similar between the two types of data collection (here represented by datasets 1 and 2); individual participants add very limited additional information after circa *n* = 40. Moreover, increasing the sample size by larger increments increased common analysis metrics (i.e., NSS and AUC) by a similar percentage. For example, between *n* = ten and *n* = 30, AUC increased by 3.8% and 3.1% for datasets 1 and 2, respectively (Tables 2 and 3, highlighted in blue).

Despite the fact that datasets 1 and 2 showed similar saturation curves, increasing the sample size by a single or multiple participants in dataset 1 was associated with a relatively greater amount of variance than in dataset 2, meaning that the added value per participant was less predictable. It is likely that each of the three factors that distinguish the datasets (lower data quality, limited monitoring of participants, using a single complex image) contributes to the lower predictability of information gain per participant in dataset 1 than in dataset 2. That is to say, studies which are most similar to dataset 1 may saturate *more* quickly, *equally* quickly, or *less* quickly than studies most similar to dataset 2 – but this is difficult to estimate in advance. As such, when using a setup which is most similar to dataset 1, researchers should consider the risk of being ’unlucky’ and collecting several participants who do not contribute to overall information gain. However, when using a setup similar to dataset 1, the (time-, monetary-, ethical-) cost per participant is generally lower than setups similar to dataset 2 – and collecting extra participants to avert those unlucky samples may therefore be proportional to the added cost.

Moreover, our results reaffirm that the type of distribution map analysis matters (cf. e.g., Le Meur & Baccino, 2013). NSS tends to keep increasing as long as large enough sample size increments are added (e.g., from 300 to 600; Table 1), whereas AUC by definition asymptotes (i.e., reaches a maximum). Given the unbounded nature of NSS, it remains sensitive to distribution map differences at very large sample sizes (such as when comparing large groups; e.g., Strauch et al., 2023), whereas AUC only marginally increased after medium-to-large sample sizes in both datasets (i.e., *n* > 30). Especially when using smaller samples (i.e., *n* $\le$ 30), AUC may be preferable over NSS because the former has a defined upper limit; a value of 1 means that two distribution maps are identical, regardless of the sample size (but see Zhao and Koch (2011); Le Meur and Baccino (2013) regarding the scaling of AUC scores). As a guideline, we therefore recommend analyzing distribution maps using bounded metrics such as AUC, especially for samples smaller than *n* = 30, but possibly also for samples larger than that. It is feasible to use NSS or other unbounded metrics for especially large samples, but keep in mind that NSS has no upper bound and it can therefore be difficult to interpret the similarity of two distribution maps.

For AOI-based analyses, the to-be-expected variance for each metric and each AOI decreases rapidly up until circa *n* = 40. After that, increasingly larger increments in sample size are needed to decrease variance by the same amount. Note that, although the averages differ between our selection of AOIs, the pattern of variance decrease is quite similar between different AOIs and different metrics. Given that variance differed between datasets 1 and 2 for distribution map analyses, it is likely that the overall variance will also be lower in AOI analyses when taking a data collection approach that is more similar to that of dataset 2. For these types of analyses, the amount of variance that one is willing to accept should thus be a leading factor in the selection of sample size. In particular, our guidelines in Tables 4 and 5 allow researchers to make an informed decision in the context of their available resources.

### Rules of thumb to use with care and sanity

The effects demonstrated here are guidelines on how many participants are needed to obtain reliable viewing statistics. These are to be handled with care, especially when it comes to AOI-based approaches. The definition of AOIs is a special sport. That is why we selected AOIs here that particularly differed on most dimensions (e.g., small/large, central/peripheral, more salient/less salient), but which are all human. These AOIs all compete for attention - some maybe being more salient, some being closer, some further to the current fixation point, some humans turning their face, some looking away, etc. Even though biased in the category (human) this well represents the attentional competition that many AOI researchers will be interested in, be it between depictions of humans, technical displays, learning materials, or other subregions of an image. If one is interested in testing whether this indeed generalizes across categories as we here proposed, then future work could systematically vary AOI shapes, sizes, and contents, including across a wider set of images. General patterns were overall comparable, suggesting generalizability in principle. Researchers may thus best inform their sample size for reliable metrics on the AOIs that best reflect their specific study and stimulus material. We here explicitly provide rules of thumb rather than definitive answers. Yet, all data and code are openly available, meaning that researchers can simulate the effects of different AOIs on the presented datasets in a way that best reflects their own studies and needs.

## Conclusion

In sum, we here provided several practical guidelines and reference tables to establish a *sufficient* yet *efficient* scene viewing participant sample size – for academics and industry professionals alike. These guidelines encompass examples of multiple study and analysis types, and we recommend that researchers pick a suitable sample size based on the similarity of our examples to their specific setup, resource constraints, and planned analyses.

## Supplementary Information

Below is the link to the electronic supplementary material.

Supplementary file 1 (pdf 989 KB)

## Data Availability

All data can be retrieved from the Open Science Framework: https://osf.io/sk4fr/.

## Code Availability

All analysis code can be retrieved from the Open Science Framework: https://osf.io/sk4fr/.

## Declarations

### Open practices statement

This research was not preregistered.
