# Supplementary material for: When is enough enough? Empirical guidelines to determine participant sample size for scene viewing studies
Source: Behav Res Methods. 2025 Jul 28;57(9):241. doi: 10.3758/s13428-025-02754-8 (PMC12304073; doi:10.3758/s13428-025-02754-8)
Supplement: Supplementary file 1 — (pdf 989 KB) [file 13428_2025_2754_MOESM1_ESM.pdf]

Supplementary Materials to: When is enough enough? Empirical guidelines to determine participant sample size for scene viewing studies

Alex J. Hoogerbrugge[1], Ignace T. C. Hooge[1], Roy S. Hessels[1], and Christoph Strauch[1,*]

[1]Experimental Psychology, Helmholtz Institute, Utrecht University, The Netherlands
[*]Corresponding author: c.strauch@uu.nl, Heidelberglaan 1, 3584 CS, Utrecht, The Netherlands

Image categories

We report the outcomes for dataset 2, split per image category. The bootstrapping procedure was run per image category subset. NSS and AUC scores were computed per image, using gaze data of all participants who viewed that image (n = 84 up to 114). The reported scores were obtained by averaging across all of those images within each subset. The view count for natural scenes (64 images) was between 107 and 114. The view count for urban scenes (64 images) was between 88 and 113. The view count for fractals (58 images) was between 85 and 90. The view count for pink noise (63 images) was between 84 and 90.

Supplementary Table 1: Sample size required for 5% relative increase (NSS and AUC) from the previous sample size, starting at n = 1.

**Fractal**				**Natural**				**Noise**				**Urban**			
AUC		NSS		AUC		NSS		AUC		NSS		AUC		NSS	
n	value	n	value	n	value	n	value	n	value	n	value	n	value	n	value
1	0.67	1	0.74	1	0.66	1	0.61	1	0.65	1	0.53	1	0.68	1	0.83
2	0.70	2	0.95	3	0.70	2	0.79	4	0.69	2	0.71	2	0.72	2	1.03
5	0.74	3	1.08	8	0.74	3	0.89	12	0.73	3	0.80	5	0.76	3	1.14
19	0.78	4	1.15	> 42	0.77	4	0.96	> 42	0.76	4	0.89	20	0.80	4	1.22
> 42	0.82	6	1.24			6	1.05			6	0.97	> 42	0.84	6	1.33
		8	1.31			9	1.13			8	1.04			9	1.40
		12	1.38			13	1.18			11	1.10			15	1.48
		20	1.45			22	1.24			16	1.16			35	1.56
		> 42	1.52			> 42	1.30			24	1.22			> 42	1.64
										> 42	1.28				

Figure 1.pdf

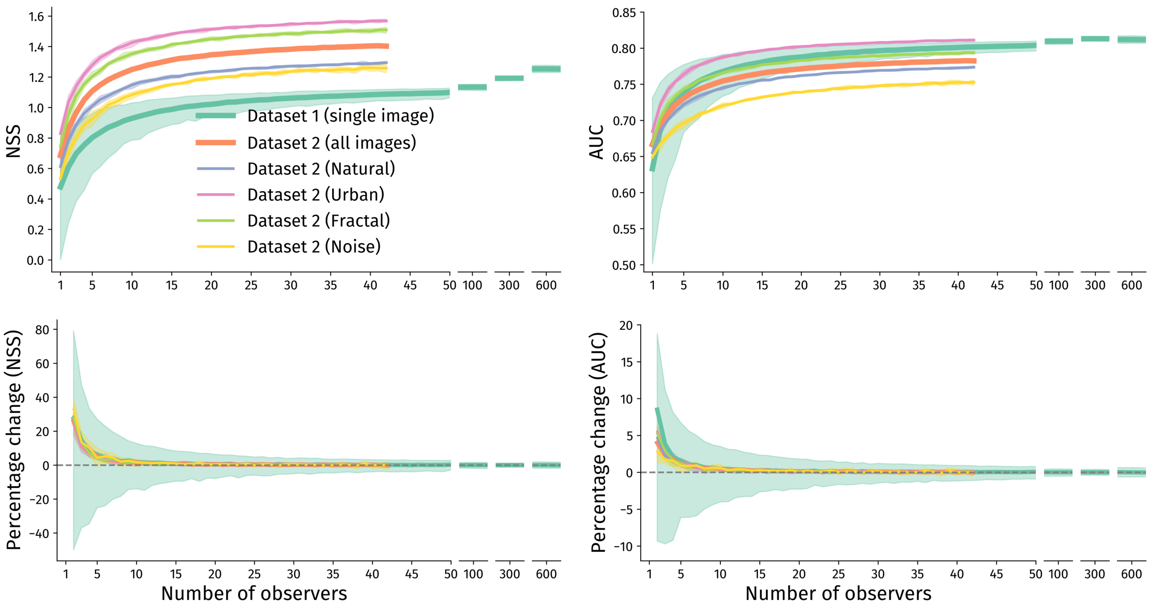

Supplementary Figure 1: Comparisons of gaze distribution maps for different sample sizes. **Top**: Distribution map similarity metrics (NSS: Normalized Scanpath Saliency; AUC: Area-Under-the-Curve) as a result of sample size compared to a 'benchmark' of all other participants. **Bottom**: Percentage change in distribution map similarity metrics per additional participant (n compared to n - 1). Dataset 1 was bootstrapped 1,000 times; dataset 2 was bootstrapped 10 times due to lower inter-individual variability. Solid lines indicate the average value, shaded areas denote the 95% range of bootstrapped values.

Supplementary Table 2: Cross-reference table. NSS and AUC percentage change between sample size increments in dataset 2 (multiple images; natural scenes).

Dataset 2 (natural scenes): NSS

	3	5	10	15	20	25	30	35	40
1	45.2	65.0	87.4	96.5	102.0	105.3	107.8	109.1	110.3
3		13.6	29.1	35.3	39.1	41.4	43.1	44.0	44.8
5			13.6	19.1	22.5	24.5	26.0	26.8	27.5
10				4.8	7.8	9.5	10.9	11.5	12.2
15					2.8	4.5	5.7	6.4	7.0
20						1.6	2.9	3.5	4.1
25							1.2	1.8	2.4
30								0.6	1.2
35									0.6

Dataset 2 (natural scenes): AUC

	3	5	10	15	20	25	30	35	40
1	7.0	10.2	13.8	15.4	16.3	17.0	17.4	17.7	18.0
3		3.0	6.3	7.9	8.8	9.4	9.8	10.0	10.3
5			3.2	4.7	5.6	6.2	6.6	6.8	7.0
10				1.4	2.3	2.8	3.2	3.5	3.7
15					0.8	1.4	1.8	2.0	2.2
20						0.5	0.9	1.2	1.4
25							0.4	0.6	0.8
30								0.2	0.5
35									0.2

Supplementary Table 3: Cross-reference table. NSS and AUC percentage change between sample size increments in dataset 2 (multiple images; urban scenes).

Dataset 2 (urban scenes): NSS

	3	5	10	15	20	25	30	35	40
1	37.8	54.2	72.0	78.9	82.5	84.7	86.9	88.0	89.1
3		12.0	24.8	29.8	32.5	34.1	35.6	36.5	37.2
5			11.5	16.0	18.4	19.8	21.2	21.9	22.6
10				4.0	6.2	7.4	8.7	9.3	9.9
15					2.1	3.3	4.5	5.1	5.7
20						1.2	2.4	3.0	3.6
25							1.2	1.8	2.4
30								0.6	1.2
35									0.6

Dataset 2 (urban scenes): AUC

	3	5	10	15	20	25	30	35	40
1	8.4	11.6	15.0	16.4	17.2	17.7	18.0	18.3	18.5
3		2.9	6.1	7.4	8.1	8.5	8.8	9.1	9.3
5			3.1	4.3	5.0	5.5	5.8	6.0	6.2
10				1.2	1.9	2.3	2.6	2.8	3.0
15					0.7	1.1	1.4	1.6	1.8
20						0.4	0.7	0.9	1.1
25							0.3	0.5	0.7
30								0.2	0.4
35									0.2

Supplementary Table 4: Cross-reference table. NSS and AUC percentage change between sample size increments in dataset 2 (multiple images; fractal images).

Dataset 2 (fractal images): NSS

	3	5	10	15	20	25	30	35	40
1	44.6	61.7	81.7	89.9	94.7	97.5	99.5	100.9	101.7
3		11.9	25.7	31.4	34.7	36.6	38.0	39.0	39.5
5			12.4	17.4	20.4	22.1	23.4	24.2	24.7
10				4.5	7.1	8.7	9.8	10.6	11.0
15					2.5	4.0	5.0	5.8	6.2
20						1.4	2.5	3.2	3.6
25							1.0	1.7	2.1
30								0.7	1.1
35									0.4

Dataset 2 (fractal images): AUC

	3	5	10	15	20	25	30	35	40
1	8.3	11.5	14.9	16.6	17.5	18.1	18.4	18.7	18.9
3		2.9	6.1	7.6	8.5	9.0	9.3	9.6	9.8
5			3.1	4.6	5.4	5.9	6.2	6.5	6.7
10				1.5	2.2	2.7	3.0	3.3	3.5
15					0.8	1.3	1.6	1.8	2.0
20						0.5	0.8	1.1	1.2
25							0.3	0.6	0.7
30								0.3	0.4
35									0.2

Supplementary Table 5: Cross-reference table. NSS and AUC percentage change between sample size increments in dataset 2 (multiple images; pink noise images).

Dataset 2 (pink noise images): NSS

	3	5	10	15	20	25	30	35	40
1	50.8	73.6	103.4	116.0	123.4	127.9	132.1	133.3	136.5
3		15.1	34.9	43.3	48.1	51.1	53.9	54.7	56.9
5			17.1	24.4	28.7	31.2	33.6	34.4	36.2
10				6.2	9.9	12.0	14.1	14.7	16.3
15					3.4	5.5	7.4	8.0	9.5
20						2.0	3.9	4.4	5.9
25							1.8	2.4	3.8
30								0.6	1.9
35									1.4

Dataset 2 (pink noise images): AUC

	3	5	10	15	20	25	30	35	40
1	4.6	7.2	11.0	12.8	13.8	14.6	15.2	15.5	15.9
3		2.5	6.2	7.8	8.8	9.6	10.2	10.4	10.9
5			3.6	5.2	6.2	6.9	7.5	7.7	8.1
10				1.6	2.5	3.2	3.8	4.0	4.4
15					0.9	1.6	2.2	2.4	2.8
20						0.7	1.2	1.5	1.9
25							0.5	0.8	1.1
30								0.2	0.6
35									0.4
